# N-Acetyltransferase-2 (NAT2) phenotype is influenced by genotype-environment interaction in Ethiopians

**DOI:** 10.1007/s00228-018-2448-y

**Published:** 2018-03-27

**Authors:** Eleni Aklillu, Juan Antonio Carrillo, Eyasu Makonnen, Leif Bertilsson, Natasa Djordjevic

**Affiliations:** 1 Division of Clinical Pharmacology, Department of Laboratory Medicine, Karolinska Institutet, Karolinska University Hospital, Huddinge C1:68, SE-141 86 Stockholm, Sweden; 2 Division of Clinical Pharmacology, Department of Medical and Surgical Therapeutics, Medical School, University of Extremadura, Badajoz, Spain; 3 Department of Pharmacology, College of Health Sciences, Addis Ababa University, Addis Ababa, Ethiopia; 4 Department of Pharmacology and Toxicology, Faculty of Medical Sciences, University of Kragujevac, Svetozara Markovica 69, Kragujevac, 34 000 Serbia

**Keywords:** NAT2 Ethiopians, Phenotype, Genotype, Environment, Gene-environment interactions, Isoniazid

## Abstract

**Background and objectives:**

N-acetyltransferase 2 (NAT2) metabolize several drugs including isoniazid. We investigated the effect of genotype, geographical difference, and smoking habit on NAT2 phenotype in Ethiopians.

**Methods:**

Genotyping for NAT2 191G > A, 341 T > C, 590G > A, and 857G > A was performed in 163 unrelated healthy Ethiopians (85 living in Ethiopia and 78 living in Sweden). The NAT2 phenotype was determined using caffeine as a probe and log AFMU/(AFMU + 1X + 1 U) urinary metabolic ratio (MR) as an index.

**Results:**

The frequencies of NAT2*4, *5, *6, *7, and *14 haplotypes were 14.1, 48.5, 30.1, 5.5, and 1.8%, respectively. The frequencies of rapid (NAT2*4/*4), intermediate (heterozygous *4), and slow (no *4 allele) acetylator genotypes were 1.8, 24.6, and 73.6%, respectively. The distribution NAT2 MR was bimodal with 70% being phenotypically slow acetylators. NAT2 genotype (*p* < 0.0001) and country of residence (*p* = 0.004) independently predicted NAT2 phenotype. Controlling for the effect of genotype, ethnic Ethiopians living in Ethiopia had significantly higher NAT2 MR than those living in Sweden (*p* = 0.006). NAT2 genotype-phenotype concordance rate was 75%. Distinct country-of-residence-based genotype-phenotype discordance was observed. The proportion of phenotypically determined rapid acetylators was significantly higher and slow acetylators was lower in Ethiopians living in Ethiopia (39% rapid, 61% slow) than in Sweden (20% rapid, 80% slow). Sex and smoking had no significant effect on NAT2 MR.

**Conclusions:**

We report a high prevalence of NAT 2 slow acetylators in Ethiopians and a conditional NAT2 genotype-phenotype discordance implicating a partial phenotype conversion and metabolic adaptation. Gene-environment interactions regulate NAT2 phenotype.

## Introduction

N-acetyltransferase 2 (NAT2), a phase II drug metabolizing enzyme, catalyzes detoxification of common carcinogens and acetylation of numerous clinically used drugs such as isoniazid, sulfonamides, procainamide, and hydralazines [1]. NAT2 acetylation capacity exhibits significant inter-individual and between-population variability, partly due to pharmacogenetic variations [2, 3]. NAT2 gene is highly polymorphic with more than hundred variant alleles described so far. A four-SNP genotype panel of 341 T > C (rs1801280), 590G > A (rs1799930), 857G > A (rs1799931), and 191G > A (rs1801279) is recommended for reliable estimation of acetylator phenotype [4–6].

NAT2 genetic diversity and acetylation phenotype are considered to be shaped by balancing and/or directional selection [7]. Adaptive evolution of NAT2 to the environment including dietary lifestyle is suggested based on a comprehensive worldwide NAT2 genetic analysis whose patterns of diversity made it plausible that slow-acetylating variants are at a weak selective pressure [8–12]. Slow acetylator phenotypes were selectively favored in populations relying on reduced folate supply, whereas the fast acetylators were neutral or even advantageous in the presence of folate-rich diets [10, 11]. Factors such as epigenetics, diet, or herbal medications are suggested as a possible cause for NAT2 genotype-phenotype discordance reported by various studies [13–19]. Muscat et al. reported that carrot and grapefruit consumption is related to NAT2 activity [20]. Recent studies highlight dietary lifestyles as a major source of variation in microbiome [21], and gut microbiota influences liver gene expression and xenobiotic metabolism [22, 23]. Consequently, environmental factors such as dietary lifestyle may cause genotype-phenotype discordance [2, 8, 24]. Individuals from the same ethnic group but living in different geographic location may respond differently to acetylated drugs. However, this hypothesis has not been investigated so far.

Isoniazid, a potent anti-tuberculosis drug, is metabolized by genetically polymorphic enzyme NAT2, and its plasma concentration is predictive of pulmonary tuberculosis outcomes [25]. Isoniazid plasma exposure, efficacy, and toxicity display wide inter-individual variability. Sub-therapeutic plasma exposure of anti-tuberculosis drugs in rapid acetylators may cause selection of resistant Mycobacterium tuberculosis strains and treatment failure. On the other hand, higher isoniazid exposure in slow NAT2 acetylates is a risk factor for anti-TB drug-induced liver toxicity [26–28]. NAT2 genotype-based isoniazid dose modification for TB personalized therapy is suggested [29–31], and genotype-guided regimen is reported to reduce isoniazid-induced liver injury and early treatment failure [32].

Globally, Ethiopia is listed among the 22 high TB burden, and one of the 30 high TB/HIV and multidrug-resistant tuberculosis (MDR-TB) burden countries, ranking third in sub-Saharan Africa [33]. With the current continuing threat of MDR-TB in Ethiopia and the increasing scale of isoniazid use for TB treatment and prevention in the country, identifying factors influencing NAT2 metabolic capacity may improve TB prevention and treatment success. Although pharmacogenetics of certain drug metabolizing enzymes and transporter proteins is well investigated [34–38], so far, there are no comprehensive studies on factors influencing NAT2 phenotype in Ethiopians. Therefore, objectives of the present study were to investigate NAT2 genotype and phenotype correlation, as well as to determine the effect of genotype, environment, sex, and smoking habit on NAT2 phenotype in Ethiopians.

## Material and methods

### Study participants

A total of 163 healthy unrelated adult Ethiopians participated in this study (85 living in Ethiopia and 78 living in Sweden [39–41]. Participants from Sweden include subjects who were adopted when they were small children by Swedish parents (*n* = 11), who lived in Sweden for more than 10 years (*n* = 30), 5–10 years (*n* = 28), and 3–5 years (*n* = 9). The study was approved by the Human Ethics Committees at Huddinge University Hospital, Karolinska Institutet, Stockholm, Sweden and the National Ethics committees at Ethiopian Science and Technology Commission, Addis Ababa, Ethiopia.

### NAT2 Phenotyping

NAT2 activity was determined in 134 subjects, using caffeine as a probe drug. All study participants refrained from taking any caffeine-containing beverage such as coffee, tea, Coca-Cola, and chocolate for at least 24 h before and throughout the study period. Study participants received a 100-mg oral dose of caffeine (Koffein; ACO AB, Helsingborg, Sweden) after emptying their bladder before bedtime, and 0 to 8 h urine was collected. The urine volume and pH were measured, pH was adjusted to 3.5 with 0.1 M HCl, and 20-ml aliquots was stored at − 20 °C until analysis. Molar concentrations of AFMU (5-acetylamino-6-formylamino-3-methyluracil), 1X (1-methylxanthine), and 1U (1-methyluric acid) in urine samples were determined using high-performance liquid chromatography as described previously [42]. Standard curves were made in the range of 5.5 to 221 μmol for AFMU; 2.8 to 271 μmol for 1X and 2.6 to 247 μmol for 1U (*r* > 0.99). The lower limit of quantitation was 5 μmol for AFMU and 2.5 μmol for 1X and 1U. A set of three concentrations of quality control samples in duplicate were incorporated into each run as follows: 13.2, 33.2, and 79.6 μmol for AFMU; 18.1, 45.1, and 108.4 μmol for 1X; and 16.5, 41.2, and 98.9 μmol for 1U. Thus, inter-day and intra-assay coefficient of variation (CV) was less than 10% at all concentration levels and for all metabolites, and the accuracy was between 96 and 105%. NAT2 phenotype was assessed using AFMU/(AFMU + 1X + 1 U) ratio as an index [3, 13].

### NAT2 genotyping

Venous blood was collected in EDTA-containing vacutainer tube, and genomic DNA was extracted from whole blood samples using the QIAamp DNA Mini Kit (QIAGEN GmbH, Hilden, Germany). Genotyping for *NAT2* gene polymorphisms 341 T > C (rs1801280), 590G > A (rs1799930), 857G > A (rs1799931), and 191G > A (rs1801279), i.e., the signature SNPs for *5, *6, *7, and *14 alleles, respectively, was performed using allele-specific PCR-RFLP as described previously [43]. The PCR reactions were performed on a GeneAmp PCR System 2700 (Applied Biosystems, Foster City, California). PCR products were visualized by gel electrophoresis on a 1.2 or 2% agarose gel stained with ethidium bromide.

### Statistical analysis

Haplotype analysis and frequency calculations were carried out using the population genetic software program Arlequin, version 3.11 (http://cmpg.unibe.ch/software/arlequin3). Statistical analyses were performed using Statistica, version 13 (StatSoft Inc, Tulsa, OK, USA), and a *p* value < 0.05 was considered significant. Chi-square test was used to assess the Hardy-Weinberg equilibrium, as well as to compare genotype, haplotypes, and acetylator phenotype frequencies between the two study groups. The AFMU/(AFMU + 1X + 1 U) ratio was log-transformed before statistical analyses. Normality of data distribution was assessed by the Shapiro-Wilk test and normal probability plot. Non-linear regression analysis was used to evaluate the effects of environment, sex, and cigarette smoking on NAT2 phenotype.

## Results

### NAT2 acetylator phenotype

**Fig. 1:**
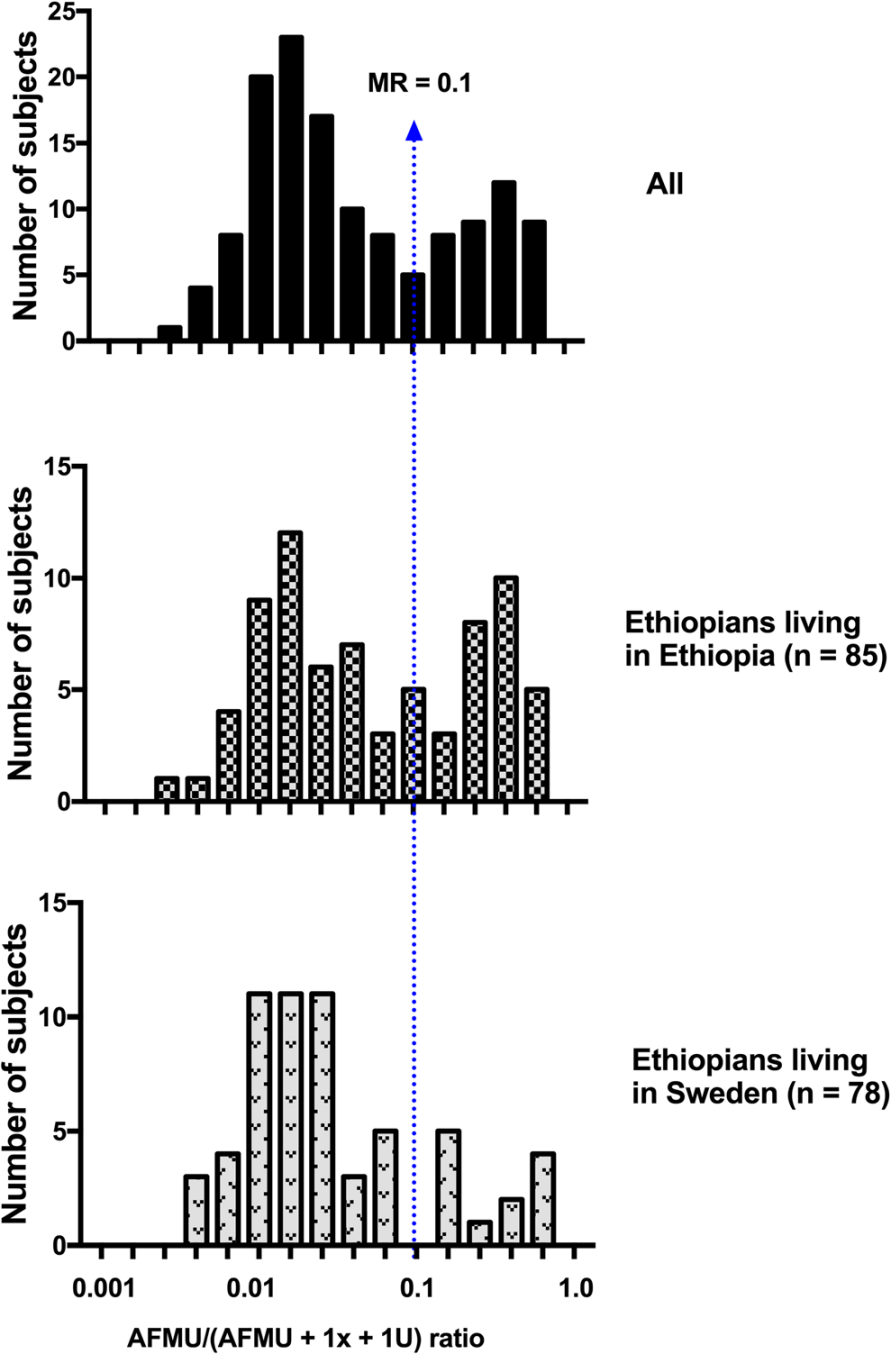
The frequency distributions of the log AFMU/(AFMU + 1X + 1 U) ratio in all ethnic Ethiopians and stratified by country of residence. The arrows indicate the anti-mode at 0.1

**Fig. 2:**
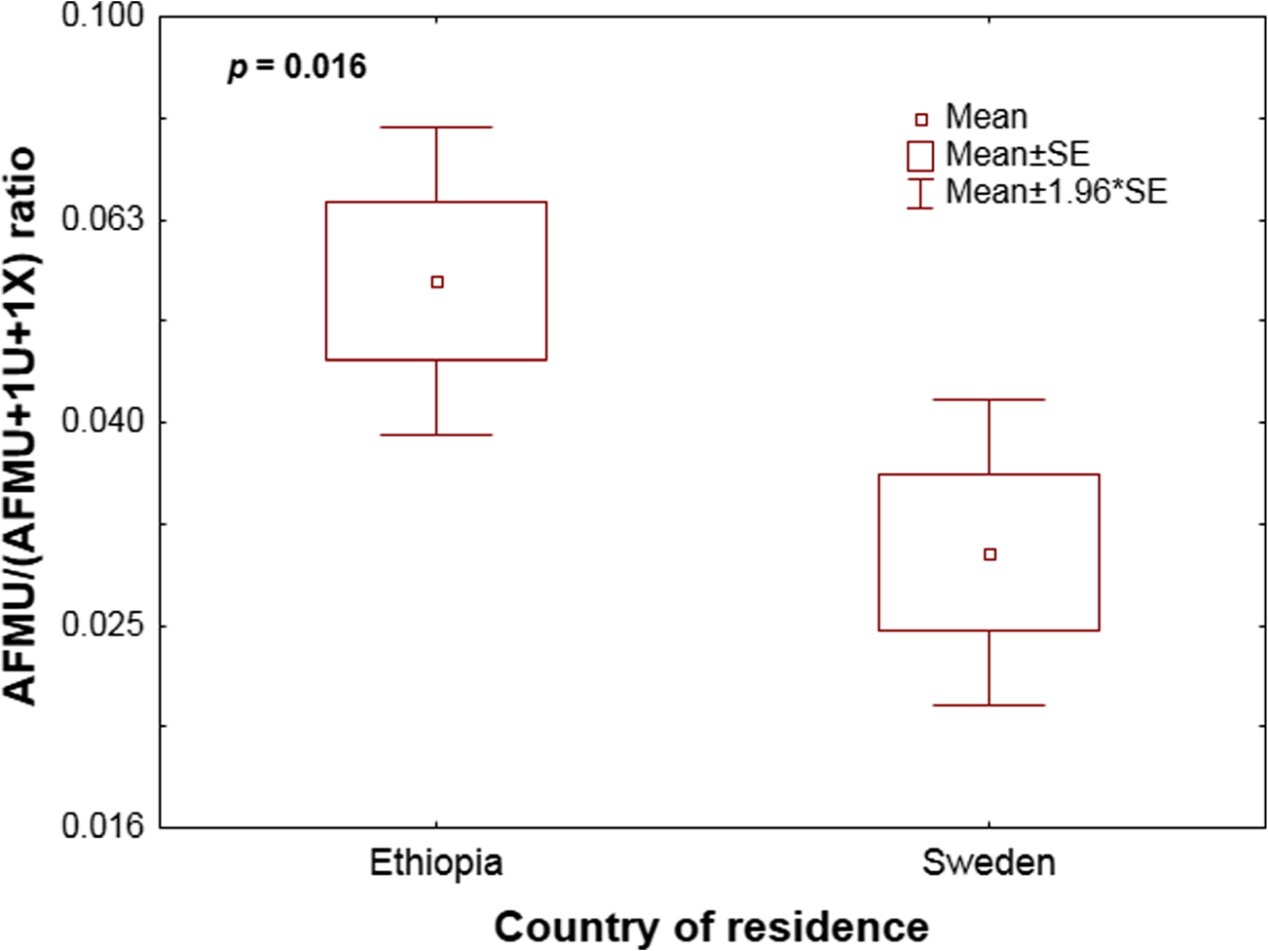
Comparison of mean log AFMU/(AFMU + 1X + 1 U) ratio between Ethiopians living in Ethiopia versus Ethiopians living in Sweden using independent *t* test

Frequency distribution of log AFMU/(AFMU + 1X + 1 U) urinary metabolic ratio in all study participants and stratified by country of residence is presented in Fig. 1. Nor mality of log NAT2 MR distribution was assessed the by Shapiro-Wilk test and normal probability plot. A bimodal frequency distribution of urinary NAT2 metabolic ratio was observed (SW-W = 0.93, *p* < 0.0001), and ranged from 0.003 to 0.743 (median 0.025; IQR 0.013; 0.176). Probit plot and regression analysis was used to identify the anti-mode cut-off value to classify slow and rapid acetylators as described previously [44, 45]. Using an anti-mode value of 0.1 as a cutoff, which was common for both study populations, 70% of the subjects were classified as phenotypically slow acetylators. The mean log AFMU/(AFMU + 1X + 1 U) urinary metabolic ratio was significantly higher in Ethiopians living in Ethiopia than those living in Sweden (independent *t* test, *p* = 0,016, geometric mean ratio = 1.86, 95% CI of the ratio: 1.13 to 3.05, Fig. 2).

### NAT2 genotype

NAT2 genotype and inferred acetylation status was done following the recommended 4-SNP genotype panel of 341 T > C (rs1801280), 590G > A (rs1799930), 857G > A (rs1799931), and 191G > A (rs1801279) [4, 5]. The frequencies of NAT2*4, *5, *6, *7, and *14 haplotypes in Ethiopian subjects living in Ethiopia or Sweden did not differ significantly (*p* > 0.05, Table 1). The overall frequencies of NAT2*4, *5, *6, *7, and *14 haplotypes in Ethiopians were 14.1, 48.5, 30.1, 5.5, and 1.8%, respectively, with no significant difference between the two study groups. Subjects homozygous wild-type for all SNPs were classified as rapid (*4), those heterozygous for any one of the SNPs were classified intermediate, and those homozygous for one or more SNPs or heterozygous for two or more SNPs were classified slow acetylators. The overall frequencies of rapid (NAT2*4/*4), intermediate (NAT2*4/*5, *4/*6, *4/*7, or *4/*14) and slow (NAT2*5/*5, *5/*6, *5/*7, *6/*6, *6/*7, *5/*14, or *6/*14) acetylator genotypes were 1.84, 24.54, and 73.62%, respectively, with no significant difference between the two study groups.

**Table 1:** Frequency distribution of NAT2 SNPs, haplotypes, and genotypes in Ethiopians living in Ethiopia or Sweden

	All	Living in Ethiopia	Living in Sweden
SNP			
rs1801280, 341 T > C	0.485 (158/326)	0.541 (92/170)	0.423 (66/156)
rs1799930, 590G > A	0.301 (98/326)	0.294 (50/170)	0.308 (48/156)
rs1799931, 857G > A	0.055 (18/326)	0.041 (7/170)	0.071 (11/156)
rs1801279, 191G > A	0.018 (6/326)	0.018 (3/170)	0.019 (3/156)
Haplotype			
NAT2*4	0.141 (46/326)	0.106 (18/170)	0.179 (28/156)
NAT2*5	0.485 (158/326)	0.541 (92/170)	0.423 (66/156)
NAT2*6	0.301 (98/326)	0.294 (50/170)	0.308 (48/156)
NAT2*7	0.055 (18/326)	0.041 (7/170)	0.071 (11/156)
NAT2*14	0.018 (6/326)	0.018 (3/170)	0.019 (3/156)
Genotype			
NAT2*4/*4	0.018 (3/163)	0.000 (0/85)	0.038 (3/78)
NAT2*4/*5	0.117 (19/163)	0.059 (5/85)	0.179 (14/78)
NAT2*4/*6	0.08 (13/163)	0.094 (8/85)	0.064 (5/78)
NAT2*4/*7	0.025 (4/163)	0.024 (2/85)	0.026 (2/78)
NAT2*5/*5	0.264 (43/163)	0.329 (28/85)	0.192 (15/78)
NAT2*5/*6	0.264 (43/163)	0.329 (28/85)	0.192 (15/78)
NAT2*5/*7	0.055 (9/163)	0.035 (3/85)	0.077 (6/78)
NAT2*6/*6	0.11 (18/163)	0.071 (6/85)	0.154 (12/78)
NAT2*6/*7	0.031 (5/163)	0.024 (2/85)	0.038 (3/78)
NAT2*4/*14	0.025 (4/163)	0.035 (3/85)	0.013 (1/78)
NAT2*5/*14	0.006 (1/163)	0.000 (0/85)	0.013 (1/78)
NAT2*6/*14	0.006 (1/163)	0.000 (0/85)	0.013 (1/78)

### NAT2 genotype-phenotype correlation

The overall all NAT2 genotype-phenotype concordance rate was 75%. NAT2 genotype accurately predicted acetylator phenotype in 100 out of 134 subjects. Concordance of acetylator status between NAT2 genotype-inferred versus measured phenotype is presented in Table 2. Although no significant difference in the NAT2 genotype between the two study groups was observed, there were significant differences in the distribution of observed acetylator phenotype (chi-square test *p* = 0.02). The proportion of phenotypically slow acetylators was higher in those living in Sweden (80%) than those living in Ethiopia (61%). On the other hand, the proportion of rapid acetylator phenotype was higher in those living in Ethiopia (39%) than those living in Sweden (20%). Distinct country of residence-based genotype-phenotype discordance was observed in 34 subjects whose genotype-deduced acetylator groups did not match with the observed acetylator phenotype based on NAT2 MR. Out of the 19 subjects who had homozygous slow genotype but had a rapid acetylator phenotype, the majority of them (16/19, 85%) were living in Ethiopia. On the contrary, 13 subjects who were heterozygous for the wild-type *4 allele (intermediate genotype) and one subjects with homozygous *4/*4 genotype displayed a slow acetylator phenotype, and majority of them (10/14, 71%) were living in Sweden.

**Table 2:** Concordance between genotype-inferred acetylator status and measured NAT2 acetylator phenotype in Ethiopians stratified by country of residence (*χ*^2^ test *p* = 0.001)

Country of residence	NAT2 genotype-inferred acetylator	NAT2 acetylator phenotype AFMU/(AFMU + 1 U + 1X) ratio		Row total
		Rapid acetylator	Slow acetylator	
Ethiopia	Rapid			
	Intermediate	13 (45%)	4 (9%)	17 (23%)
	Slow	16 (55%)	41 (91%)	57 (77%)
	Sub total	29 (39%)	45 (61%)	74
Sweden	Rapid	1 (8.3%)	1 (2%)	2 (3.3%)
	Intermediate	8 (66.7%)	9 (19%)	17 (28.3%)
	Slow	3 (25.0%)	38 (79%)	41 (68.4%)
	Sub total	12 (20%)	48 (80%)	60
All		41 (30.6%)	93 (69.4%)	134

### Factors affecting NAT2 phenotype

**Fig. 3:**
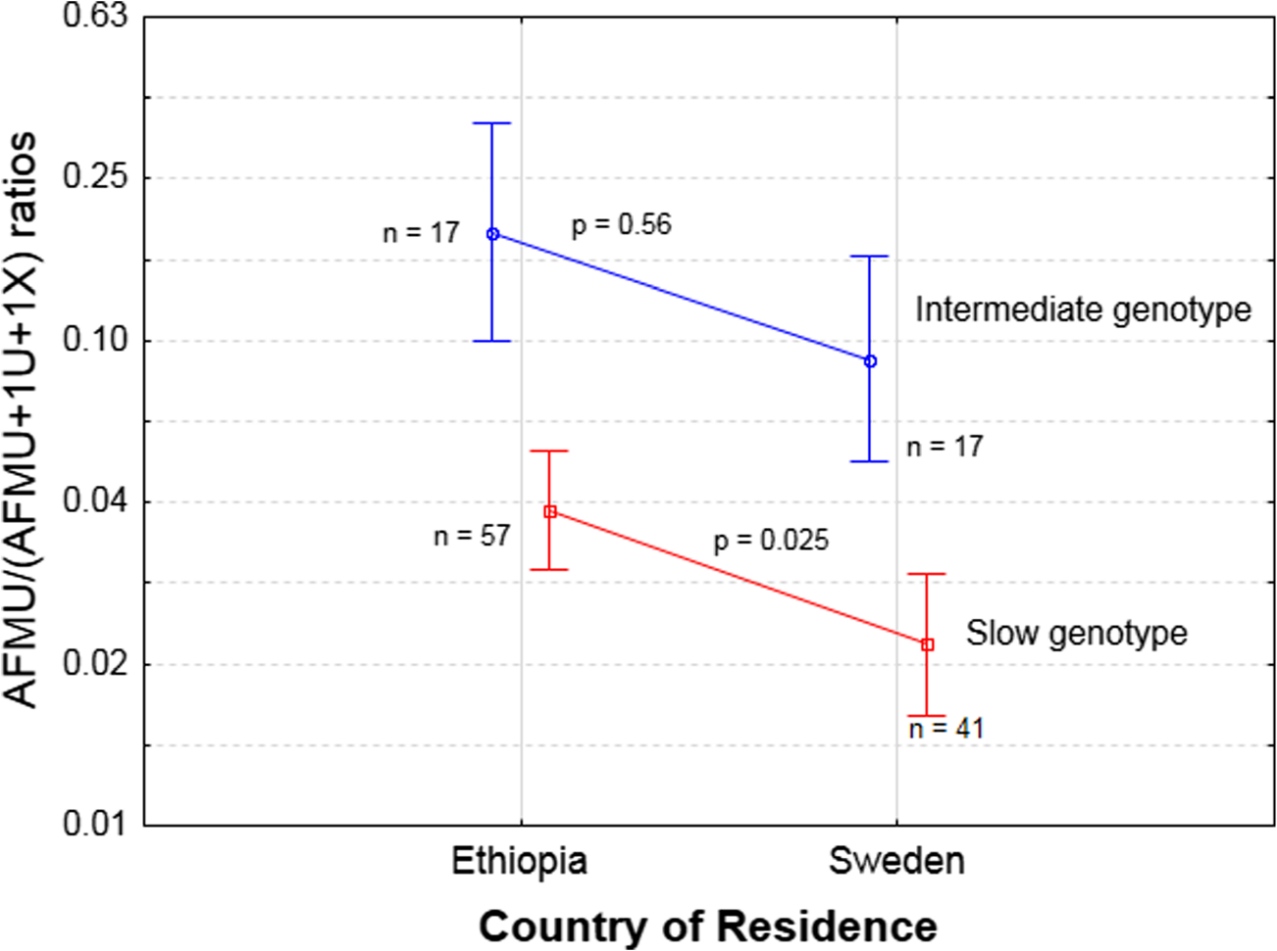
Comparison of mean NAT2 urinary metabolic ratios between Ethiopians living in Ethiopia and Ethiopian living in Sweden stratified by NAT2 genotype-inferred acetylators group as intermediate and slow group. Vertical bars denote 95% confidence interval of the mean

Both NAT2 genotype (*p* < 0.0001) and country of residence (*p* = 0.004) independently and significantly influenced NAT2 MR. Non-linear regression analysis indicated a significant influence of *NAT2* genotype on phenotype (*p* = 0.002). Controlling for the genotype effect, significantly higher NAT2 MR (*p* = 0.006) was observed in Ethiopians living in Ethiopia. Having the same genotype-inferred acetylator status (slow and intermediate acetylators) the mean log NAT2 MR was much higher in those living in Ethiopia than in Sweden. Factorial ANOVA indicated that both NAT2 genotype and country of residence as significant predictors of NAT2 phenotype independently. Post-hoc analysis using the Bonferroni correction indicated that slow acetylators living in Ethiopia had a significantly higher mean MR than those living in Sweden (*p* = 0.025, Fig. 3). A similar but non-significant trend was observed among intermediate acetylators (heterozygous for *4 allele) which were few (Fig. 3). None of those living in Ethiopia were homozygous for *4 (Table 1). Sex (*p* = 0.10) and cigarette smoking (*p* = 0.57) did not affect NAT2 phenotype.

## Discussion

In the present study, we investigated NAT2 genotype-phenotype correlations and the impact of sex, age, and geographic differences on NAT2 phenotype in Ethiopians. Our main findings include (i) a high but not complete concordance between NAT2 genotype and phenotype, (ii) a high prevalence of slow acetylators in Ethiopians, and (iii) despite a similar NAT2 genotype frequency a significantly different proportion of NAT2 acetylation phenotypes between ethnic Ethiopians living in Ethiopia or Sweden. As to our best knowledge, this is the first study to investigate NAT2 genotype-phenotype correlation in same ethnic group but live in different geographic location.

The inference of acetylation phenotype from *NAT2* genotype is widely accepted, and the phenotype prediction accuracy can reach up to 100% [46]. However, lack of complete genotype-phenotype concordance is not uncommon [13–19]. Using caffeine as a probe and NAT2 gene sequencing for genotyping, a high (86%) genotype-phenotype discordance rate is reported in south-eastern Asian immigrants living in the USA [15]. The authors suggested environmental factors such as diet or unreported non-traditional medications as a possible cause for altered metabolic phenotype. Using dapsone as a probe drug, 18% discordance rate between genotype-deduced and determined acetylator phenotype in HIV patients is reported [18]. A 49.1% NAT2 genotype-phenotype discordance rate in tuberculosis-infected Ethiopian patients is reported previously [19].

Using caffeine and urinary AFMU/(AFMU + 1X + 1 U) metabolite ratio as the current state of the art method of determining NAT2 activity in vivo [6, 47], our results confirm a high prevalence (70%) of slow acetylators in an Ethiopian population. The use of caffeine as a probe drug for NAT2 phenotyping has been questioned, owing to the intrinsic instability of AFMU which deformylates to AAMU. However, kinetics studies have demonstrated that AFMU is a relatively stable compound over the pH range of 2.0–8.0 at 24 °C and the rate of AFMU deformylation in urine is negligible over a relatively long period of time during sample storage at pH 3.5 and − 20 °C [48], which we applied in our method. Although previous conversion of AFMU to AAMU may appear as an advantage for NAT2 phenotyping, the AFMU/(AFMU + 1X + 1 U) ratio, which is unaffected by xanthine oxidase activity, has demonstrated to be a robust and appropriate marker for NAT2 enzyme activity. The use of AFMU in both the numerator and denominator of the metabolic ratio would minimize the effect of spontaneous AFMU deformylation. In principle, both AFMU and AAMU can be used for NAT2 metabolite ratio determination. However, it seems that AAMU might not be formed solely through a nonenzymatic hydrolysis of AFMU in urine, but in vivo from another source that requires further confirmation [49].

The finding of this study confirms the expected high but not complete correlation between *NAT2* genotype and observed acetylator status in Ethiopians. Selection of NAT2 variant alleles in the present study was based on our previous comprehensive NAT2 genotype analysis in Ethiopians using gene sequencing which showed that NAT2*5, *6 and *7, and *14 as the only detected defective variant alleles [27]. The prevalence of genotype-inferred NAT2 acetylation status using gene sequencing indicated 68.7% of Ethiopians as slow acetylators, while 31.3% were rapid acetylators [27], which is similar to what is found in the present study using allele-specific PCR (70% slow, and 30% rapid acetylators). Therefore, it is unlikely that rare-defective variant alleles, which are not genotyped in the present study, as a cause for the observed partial genotype-phenotype discordance.

Interestingly, distinct country of residence-based genotype-phenotype discordance was observed. Among the 19 phenotypically rapid acetylators but genotypically slow acetylators, the majority (85%) of them were living in Ethiopia. On the other hand, among the 14 phenotypically slow acetylator, despite being carriers of the wild-type allele (*4), the majority (71%) of them were living in Sweden (Table 2). Partial and conditional conversion to phenotypically rapid acetylators despite being genotype-deduced slow acetylator in those living in Ethiopia and the occurrence of quite a contrary event in those living in Sweden may implicate phenotypic adaptation to the environment. Conditional phenotype conversion and metabolic adaptation as a result of genotype-environment interactions are reported recently [50]. Not only we observed partial phenotypic conversion but also phenotype boosting. Despite having the same genotype-deduced acetylator phenotype, higher NAT2 MR in those living in Ethiopia than in Sweden (Fig. 3) may further suggest additional environmental factors that boost NAT2 phenotype towards rapid acetylator phenotype in Ethiopia. We reported earlier a significant higher NAT2 enzyme activity in Koreans than Swedes, having the same genotype [3]. In the present study, a higher NAT2 metabolic activity in Ethiopians living in Ethiopia than those living in Sweden may indicate possible implications of environmental factors such as dietary habit. Interestingly, using the same probe drug caffeine, we reported a similar higher xanthine oxidase enzyme activity in Ethiopians living in Ethiopia than Sweden [40], but no significant influence of the geographic difference in CYP2A6 and CYP1A2 enzyme activity was noted [41, 51].

Our finding supports a possible independent influence of both the dietary habits associated with subsistence modes and the chemical environment associated with climatic zones and biomes on the evolution of NAT2 diversity in sub-Saharan African populations suggested previously [8]. Subsistence mode of a population, which determines the exposure to different substances through dietary habit or a lifestyle, can direct and adjust the metabolism of an individual to a favorable scope and level with time [8, 52]. Diet rapidly and reproducibly alters the human gut microbiome [21] and it is a major determinant of gut microbial composition, which is different between Europe and Africa. Gut microbiome plays a key role in modulating epigenetics, drug metabolism, efficacy, and toxicity, and may cause between individuals and populations variability [22, 23]. Gut microbiota influences liver gene expression and alters xenobiotic metabolism [23], and it can rapidly respond to the diversity of human dietary lifestyles [21]. Thus, unlike genetic adaptation, which requires generations to occur, it is plausible to assume that conditional and partial phenotypic adaptation of xenobiotic-metabolizing enzymes to environmental factors including drugs, dietary, and herbal constituents to be rather prompt. A recent study in animal model reported differential response to environmental factors (defined by different conditions of husbandry), to cause partial reversal of phenotypes [50]. Future genetic, epigenetic, and microbiome studies are needed to explain NAT2 genotypic and phenotypic adaptation to environmental factors including dietary and lifestyle changes.

## Conclusion

Overall, the prevalence of slow NAT2 acetylator phenotype is high in Ethiopians, but the prevalence varied depending on country of residence. NAT2 genotype and country of residence independently and significantly influence NAT2 acetylator phenotype. There is high but not complete concordance between NAT2 genotype and phenotype in Ethiopians. The observed distinct country-of-residence-based genotype-phenotype discordance may indicate a conditional and partial NAT2 phenotype conversion and metabolic adaptation to the environment. Gene-environment interactions play an important role in regulating NAT2 phenotype. Future quantitative assessment of environmental influences is needed to elucidate the extent of gene-environment interaction for NAT2 personalized medicine and genotype-based drug dosing. Our finding highlights the need for NAT2 genetic characterization in different African populations for treatment optimization targeting individual/population make-up.
